# Efficacy of nano-sized ultrafine water clusters in reducing erythema following fractionated picosecond alexandrite laser treatment: a split-face, randomized, evaluator-blinded pilot study

**DOI:** 10.1007/s10103-026-04967-5

**Published:** 2026-07-22

**Authors:** Kentaro Oku

**Affiliations:** HILLS GRACE CLINIC, Yokohama, Japan

**Keywords:** Ultrafine water clusters, Picosecond alexandrite laser, Post-laser erythema, Hemoglobin-enhanced imaging, Split-face study, PEDOT/PSS

## Abstract

Post-procedural erythema following energy-based dermatological treatments is a common concern affecting patient satisfaction and downtime. Nano-sized ultrafine water clusters (UFW), generated by conductive polymer poly(3,4-ethylenedioxythiophene)–poly(styrene sulfonate) (PEDOT/PSS) technology, represent a nanometer-scale water cluster species (average aerosol-phase particle diameter ≈ 1.4 nm). This split-face, randomized, evaluator-blinded pilot study evaluated the efficacy of UFW in reducing erythema following fractionated picosecond alexandrite laser (Fr-PSAL) treatment with a diffractive lens array. Twenty-five female subjects were assigned to a pre-treatment (*n* = 13) or post-treatment (*n* = 12) group. UFW was applied for 30 min to one randomly selected facial side; the contralateral side served as control. Two independent board-certified dermatologists, blinded to allocation, correctly identified the UFW-treated side with high concordance in both groups. In the post-treatment group, each evaluator achieved 11/12 correct identifications (91.7%, binomial *p* = 0.003); Cohen’s kappa between evaluators was 0.625 (substantial agreement). In the pre-treatment group, Evaluator 1 identified 11/13 correct (84.6%, *p* = 0.011) and Evaluator 2 identified 12/13 correct (92.3%, *p* = 0.002); Cohen’s kappa was 0.831 (almost perfect agreement). An exploratory semi-quantitative hemoglobin-enhanced image analysis provided objective support, although the strength of that support differed between the two groups. In the post-treatment group, the pre-specified primary metric (erythema density) was robustly significant (Cohen’s d = − 0.93, *p* = 0.004). In the pre-treatment group, the pre-specified primary metric (erythema area) was also significant (d = − 0.57, *p* = 0.031), but this prophylactic-arm result depended on per-subject selection of the comparison timepoint and was not significant when a fixed 6 h timepoint was used (7/13 correct, 53.8%; *p* = 0.448); it should therefore be regarded as hypothesis-generating. No adverse events were observed. These results provide the first clinical evidence that UFW effectively reduces post-laser erythema, with findings consistent with both therapeutic and prophylactic mechanisms of action.

**Supplementary Information:**

The online version contains supplementary material available at 10.1007/s10103-026-04967-5.

## Introduction

Nano-sized ultrafine water clusters (UFW), generated using conductive polymer poly(3,4-ethylenedioxythiophene)–poly(styrene sulfonate) (PEDOT/PSS) technology, represent a nanometer-scale water cluster species with distinct emission characteristics compared with conventional humidification. The PEDOT/PSS polymer absorbs ambient moisture and, upon controlled heating, releases water clusters whose size has been characterized in the aerosol phase by electrical-mobility analysis. In this measurement the airborne water clusters are charged by an americium-241 bipolar ion source, classified according to their electrical mobility in a nano-differential mobility analyzer (nano-DMA), and detected with a Faraday-cup electrometer, so that the reported value is an equivalent electrical-mobility diameter rather than a directly imaged geometric diameter; introducing the fine water shifted the positive-ion peak from 1.19 nm in ambient laboratory air to 1.36–1.46 nm, corresponding to an average mobility diameter of approximately 1.4 nm (Supplementary Material 1). Although individual water molecules retain their intrinsic dipole moment, the effective dielectric response of water can change at the nanoscale under strong confinement. For example, it has been reported that water confined between atomically flat walls with separations down to ~ 1 nm exhibits a markedly low out-of-plane dielectric constant. While this finding relates to highly confined interfacial water and does not directly correspond to free aerosol clusters, it highlights that nanoscale structuring can substantially alter the macroscopic polar response of water [1]. Such changes in effective polarity may be relevant to how water interacts with biological interfaces such as the stratum corneum. In this context, the hydration- and barrier-recovery effects of UFW, demonstrated in vitro using caffeine as a hydrophilic tracer [2] and in vivo by sustained increases in stratum corneum water content [3], are consistent with the notion that nanoscale water structuring can influence skin barrier function.

In cosmetic dermatology, minimizing post-treatment downtime while maintaining therapeutic efficacy is a critical challenge. Energy-based devices such as radiofrequency, ultrasound, and lasers commonly cause post-procedural erythema due to thermal effects. Fractionated picosecond alexandrite laser (Fr-PSAL) treatment employing a diffractive lens array (DLA) has demonstrated high tolerability and effectiveness for fine wrinkles [4], skin quality improvement [5, 6], and acne scars [7, 8]; however, post-treatment erythema remains inevitable. Reducing the degree and shortening the duration of erythema through entirely non-invasive approaches could significantly benefit patient outcomes.

To date, evidence for non-invasive erythema-reducing interventions following laser treatment has relied primarily on subjective evaluator assessment. This pilot study aimed to evaluate the efficacy and safety of UFW applied as pre- and post-treatment interventions for reducing erythema following Fr-PSAL, using blinded clinical evaluation as the primary outcome and a novel semi-quantitative hemoglobin-enhanced image analysis as an exploratory secondary outcome.

## Materials and methods

### Ethics

This study was conducted in accordance with the principles of the Declaration of Helsinki and was approved by the Shiba Palace Clinic Ethics Review Board, an independent third-party ethics committee distinct from the investigator’s affiliated institution. Written informed consent was obtained from all participants, including explicit permission for the use of their photographs for publication. 

### Subjects

Participants included female individuals aged 25–60 years who had previously experienced erythema following Fr-PSAL treatments and were not using skincare products potentially affecting erythema. No formal sample size calculation was performed for this pilot study; the sample size was based on practical feasibility.

### Devices

The UFW generator used was Windscell (Aisin Corporation, Kariya, Aichi, Japan). The device absorbs ambient humidity through its PEDOT/PSS conductive polymer layer and, upon controlled heating, releases nano-sized ultrafine water clusters through its outlet. UFW emission was operated on a 1-minute-on / 1-minute-off cycle throughout the 30-minute application period, yielding approximately 15 min of cumulative active UFW emission. Because UFW dispatch occurs by heating the PEDOT/PSS layer, warm air was also released during operation.

The fractionated picosecond alexandrite laser used was PICOSUREpro (Cynosure Lutronic, Inc., Westford, MA, USA), emitting 755 nm wavelength with picosecond pulse duration (550–750 ps, adjustable) and equipped with a diffractive lens array (DLA/FOCUS). The DLA modifies the intensity profile of the laser beam to produce a hexagonal array of high-intensity regions surrounded by a low-intensity background, inducing laser-induced optical breakdown (LIOB) within the epidermis.

### Study design and intervention

This was a split-face, randomized, evaluator-blinded pilot study. Twenty-five subjects were assigned to one of two groups:

Pre-treatment group (*n* = 13): UFW was randomly applied for 30 min to one side of the face using Windscell. The contralateral side was covered with plastic wrap and water-soaked gauze to ensure similar thermal conditions and prevent UFW penetration. Immediately after UFW application, Fr-PSAL was administered to the entire face in a single pass (8 mm spot size, 0.33 J/cm², 550 ps). Hemoglobin-enhanced images were captured with a skin analyzer Re-Beau2 (JMEC Co., Ltd., Tokyo, Japan) at three timepoints: immediately after Fr-PSAL (0 h), 1 h, and 6 h post-treatment. Images were captured from three angles: frontal, right 45°, and left 45°.

Post-treatment group (*n* = 12): Fr-PSAL was applied to the entire face in a single pass under the same parameters. Baseline hemoglobin-enhanced images were captured prior to laser treatment. Immediately afterward, UFW was randomly applied to one side for 30 min while the contralateral side was covered as described above. Post-treatment images were captured immediately after laser treatment (before UFW application) and immediately after UFW application.

The specific control condition (water-soaked gauze beneath plastic wrap) was selected on the basis of a preliminary phantom experiment conducted prior to the clinical study, in which a heated mannequin simulating skin was used to compare the surface-temperature trajectory of the UFW-treated condition (Windscell warm airflow + UFW aerosol), the clinical control condition (water-soaked gauze beneath plastic wrap), and a reference occlusion condition (plastic wrap only, without gauze). Over approximately 30 min of application, the clinical control condition tracked the UFW-treated condition closely (endpoint surface temperatures of 32.4 °C and 31.4 °C, respectively; net cooling − 5.1 °C vs. −6.3 °C), whereas the reference occlusion condition retained heat and showed a net temperature rise to 41.8 °C. These preliminary data confirmed that the clinical control condition provides approximately equivalent thermal dissipation to the UFW-treated condition and avoids the heat-trapping confound of simple occlusion. Full details of this preliminary experiment are provided in Supplementary Material 3.

### Imaging conditions

All hemoglobin-enhanced images were captured in a temperature-controlled room maintained at 24 ± 1 °C throughout the study period. Because hemoglobin-enhanced imaging is sensitive to cutaneous blood flow, which varies with ambient temperature, standardized thermal conditions were maintained to minimize temperature-dependent fluctuations in the hemoglobin signal. Subjects rested in the imaging room for at least 10 min prior to the first image capture to allow acclimatization of facial skin temperature. Consistent lighting conditions were ensured by the built-in standardized illumination system of the Re-Beau2 skin analyzer.

### Primary outcome: blinded clinical evaluation

The primary outcome was the proportion of subjects in whom the UFW-treated facial side was correctly identified by two independent, blinded, board-certified dermatologists reviewing hemoglobin-enhanced images. Correct identification was defined as the evaluator’s designation of the facial side that had actually received UFW. Each evaluator made independent judgments blinded to both treatment allocation and the other evaluator’s judgments. The correct-identification proportion in each group was tested against chance (50%) using a binomial test. Inter-rater reliability between the two evaluators was quantified using Cohen’s kappa, with the Landis-Koch (1977) benchmark used for interpretive classification [9].

### Exploratory secondary outcome: semi-quantitative image analysis

As an exploratory secondary analysis to provide objective corroboration, semi-quantitative image analysis was performed on lateral-view hemoglobin-enhanced images (right 45° for the right cheek, left 45° for the left cheek). Digital images were processed using a custom analysis pipeline implemented in Python (version 3.13) with NumPy (version 2.2) and Pillow (version 11.1). Each lateral-view image was cropped to the central 70% (width) × 80% (height) to exclude peripheral artifacts. Skin pixels were identified using a color-space mask: red channel (R) ∈ [80, 250], green channel (G) ∈ [40, 220], and R − G > − 10. The R − G channel difference served as a hemoglobin index, reflecting the relative contribution of oxyhemoglobin absorption in the red wavelength band.

To isolate clinically meaningful erythema, a fixed threshold of R − G > 50 was applied. Two complementary parameters were derived: (1) erythema density, the mean R − G value among threshold-exceeding pixels, representing the chromatic intensity of erythematous regions; and (2) erythema area, the proportion of threshold-exceeding pixels relative to total skin pixels, representing the spatial extent of erythema.

Both erythema density and erythema area were pre-specified as candidate exploratory outcome measures. The assignment of the primary exploratory metric for each treatment arm was also pre-specified on the basis of distinct mechanistic hypotheses formulated prior to data analysis. In the post-treatment group, the hypothesized therapeutic mechanism is the attenuation of vascular engorgement within already-established erythematous foci; the anticipated observable change is therefore a reduction in intensity, and erythema density was pre-specified as the primary exploratory metric. In the pre-treatment group, the hypothesized prophylactic mechanism is the attenuation of new erythematous-focus formation during laser injury; the anticipated observable change is therefore a reduction in spatial extent, and erythema area was pre-specified as the primary exploratory metric. The alternative metric within each group was pre-specified as a sensitivity analysis to probe whether the primary metric captures the biologically anticipated dimension of change.

### Statistical analysis

For the primary outcome, a binomial test assessed whether the correct identification rate significantly exceeded chance (50%). For the exploratory image analysis, the difference in the erythema metric between UFW-treated and control sides was computed for each subject. A one-sided paired t-test evaluated the directional hypothesis that the UFW-treated side exhibited greater erythema reduction. The Wilcoxon signed-rank test was used as a non-parametric confirmatory analysis. Effect size was reported as Cohen’s d. Statistical analyses were performed using SciPy (version 1.15). A p-value < 0.05 was considered statistically significant.

### Safety evaluation

An independent board-certified dermatologist evaluated subjective and objective post-treatment skin reactions.

## Results

### Subject demographics

Pre-treatment group: thirteen female subjects (ages 26–57; mean 41.9 ± 8.8 years) with Fitzpatrick skin types II (*n* = 7), III (*n* = 5), and IV (*n* = 1) completed the study. Post-treatment group: twelve female subjects (ages 26–52; mean 39.8 ± 8.2 years) with Fitzpatrick skin types II (*n* = 8), III (*n* = 3), and IV (*n* = 1) completed the study.

### Laser treatment parameters

The mean number of Fr-PSAL shots delivered per session was 2082 ± 135 (range: 1850–2310) across all subjects.

### Primary outcome: blinded clinical evaluation

**Fig. 1:**
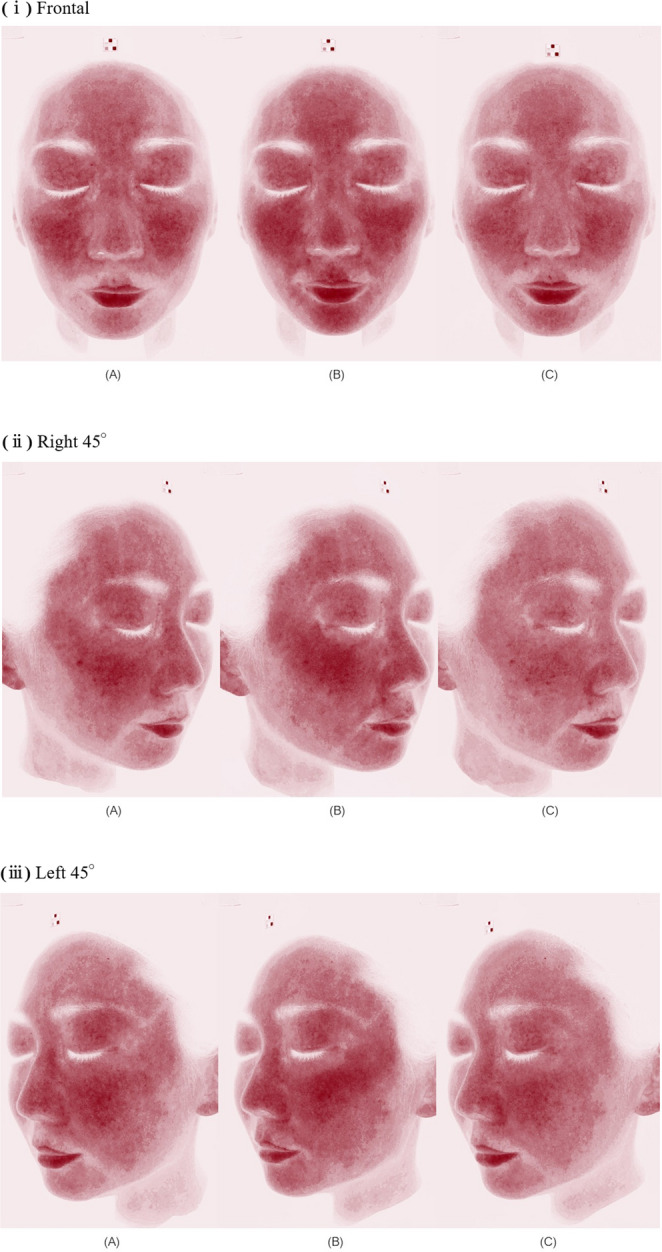
Hemoglobin-enhanced images of a 39-year-old female subject in the Post-treatment group. (ⅰ) Frontal. The right side of the face was treated with nano-sized ultrafine water clusters (UFW). In images captured immediately after UFW application, both a reduction in the extent of erythema and a decrease in erythema severity were observed on the UFW-treated side. (**A**) Before the Fr-PSAL treatment. (**B**) Immediately after the Fr-PSAL, Before the UFW application. (**C**) Immediately after the UFW application. (ⅱ) Right 45°. The right side of the face was treated with nano-sized ultrafine water clusters (UFW). In images captured immediately after UFW application, both a reduction in the extent of erythema and a decrease in erythema severity were observed on the UFW-treated side. (**A**) Before the Fr-PSAL treatment. (**B**) Immediately after the Fr-PSAL, Before the UFW application. (**C**) Immediately after the UFW application. (ⅲ) Left 45°.The right side of the face was treated with nano-sized ultrafine water clusters (UFW). In images captured immediately after UFW application, both a reduction in the extent of erythema and a decrease in erythema severity were observed on the UFW-treated side. (**A**) Before the Fr-PSAL treatment. (**B**) Immediately after the Fr-PSAL, Before the UFW application. (**C**) Immediately after the UFW application

**Fig. 2:**
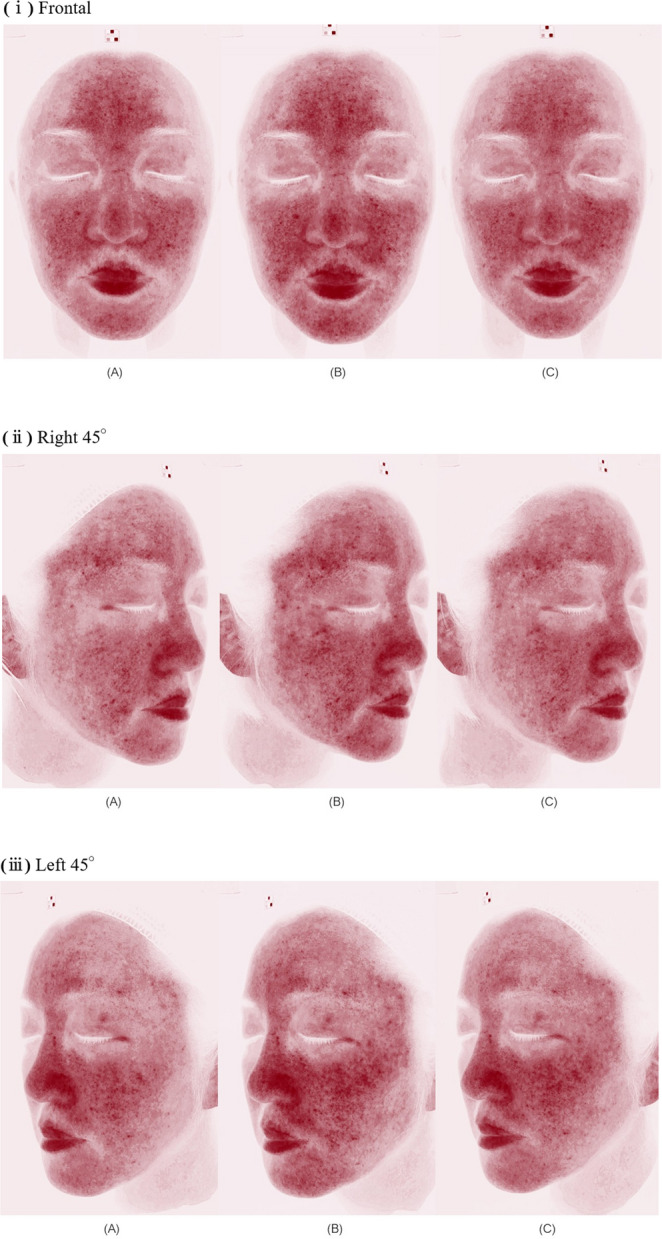
Hemoglobin-enhanced images of a 52-year-old female subject in the Post-treatment group. (ⅰ) Frontal. The right side of the face was treated with nano-sized ultrafine water clusters (UFW). On the UFW-treated side, erythema was reduced below baseline levels observed prior to laser irradiation. (**A**) Before the Fr-PSAL treatment (Baseline). (**B**) Immediately after the Fr-PSAL, Before the UFW application. (**C**) Immediately after the UFW application. (ⅱ) Right 45°.The right side of the face was treated with nano-sized ultrafine water clusters (UFW). On the UFW-treated side, erythema was reduced below baseline levels observed prior to laser irradiation. (**A**) Before the Fr-PSAL treatment (Baseline). (**B**) Immediately after the Fr-PSAL, Before the UFW application. (**C**) Immediately after the UFW application. (ⅲ) Left 45°.The right side of the face was treated with nano-sized ultrafine water clusters (UFW). On the UFW-treated side, erythema was reduced below baseline levels observed prior to laser irradiation. (**A**) Before the Fr-PSAL treatment (Baseline). (**B**) Immediately after the Fr-PSAL, Before the UFW application

**Fig. 3:**
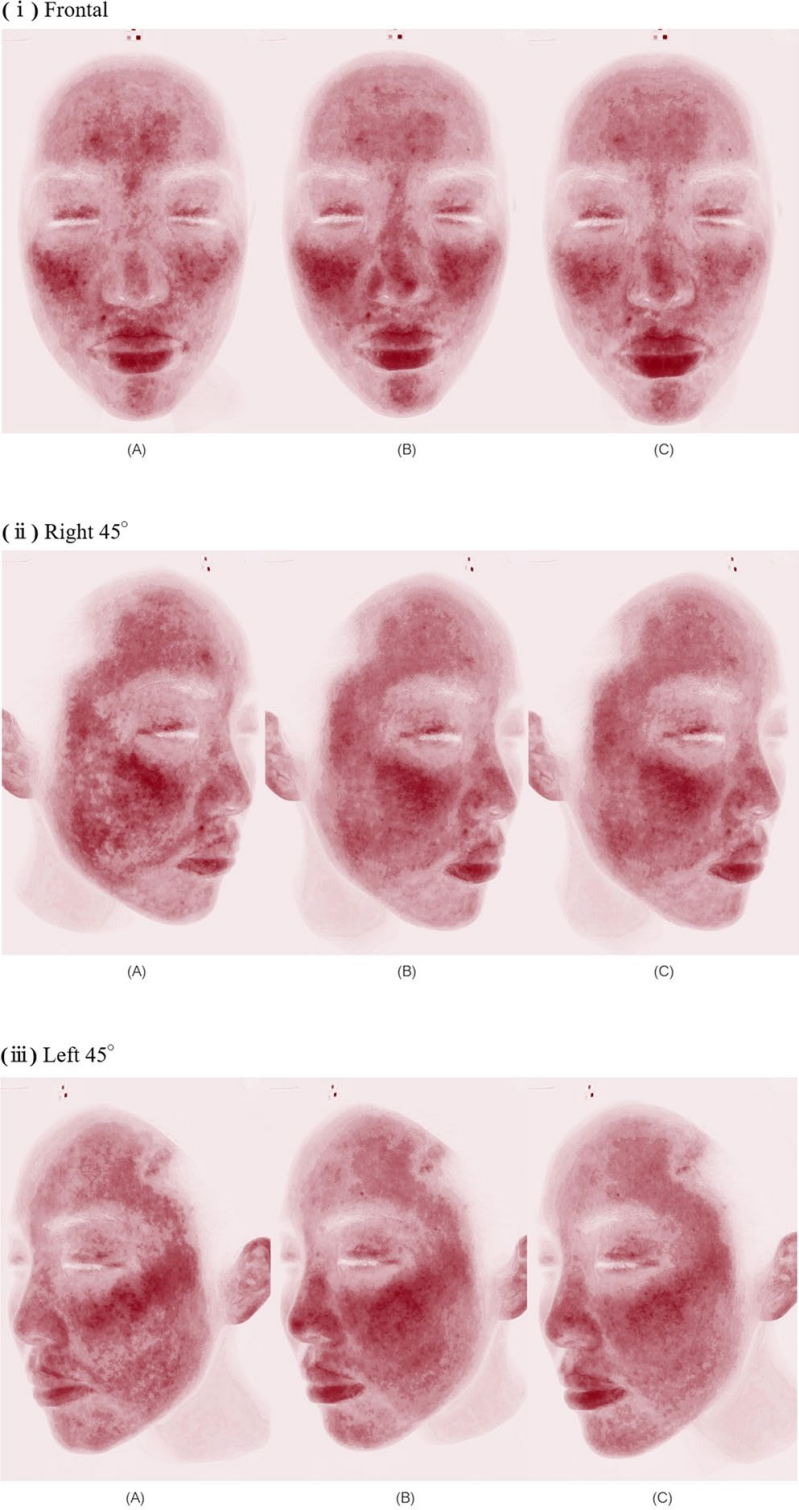
Hemoglobin-enhanced images of a 26-year-old female subject in the Pre-treatment group. (ⅰ) Frontal. The Left side of the face was treated with nano-sized ultrafine water clusters (UFW). The occurrence of erythema after Fr-PSAL irradiation was confirmed to be milder on the UFW-treated side. (**A**) Immediately after the Fr-PSAL irradiation. (**B**) 1-hour after the Fr-PSAL. (**C**) 6-hour after the Fr-PSAL. **(ⅱ)** Right 45°.The Left side of the face was treated with nano-sized ultrafine water clusters (UFW). The occurrence of erythema after Fr-PSAL irradiation was confirmed to be milder on the UFW-treated side. (**A**) Immediately after the Fr-PSAL irradiation. (**B**) 1-hour after the Fr-PSAL. (**C**) 6-hour after the Fr-PSAL. **(ⅲ)** Left 45°.The Left side of the face was treated with nano-sized ultrafine water clusters (UFW). The occurrence of erythema after Fr-PSAL irradiation was confirmed to be milder on the UFW-treated side. (**A**) Immediately after the Fr-PSAL irradiation. (**B**) 1-hour after the Fr-PSAL. (**C**) 6-hour after the Fr-PSAL

**Fig. 4:**
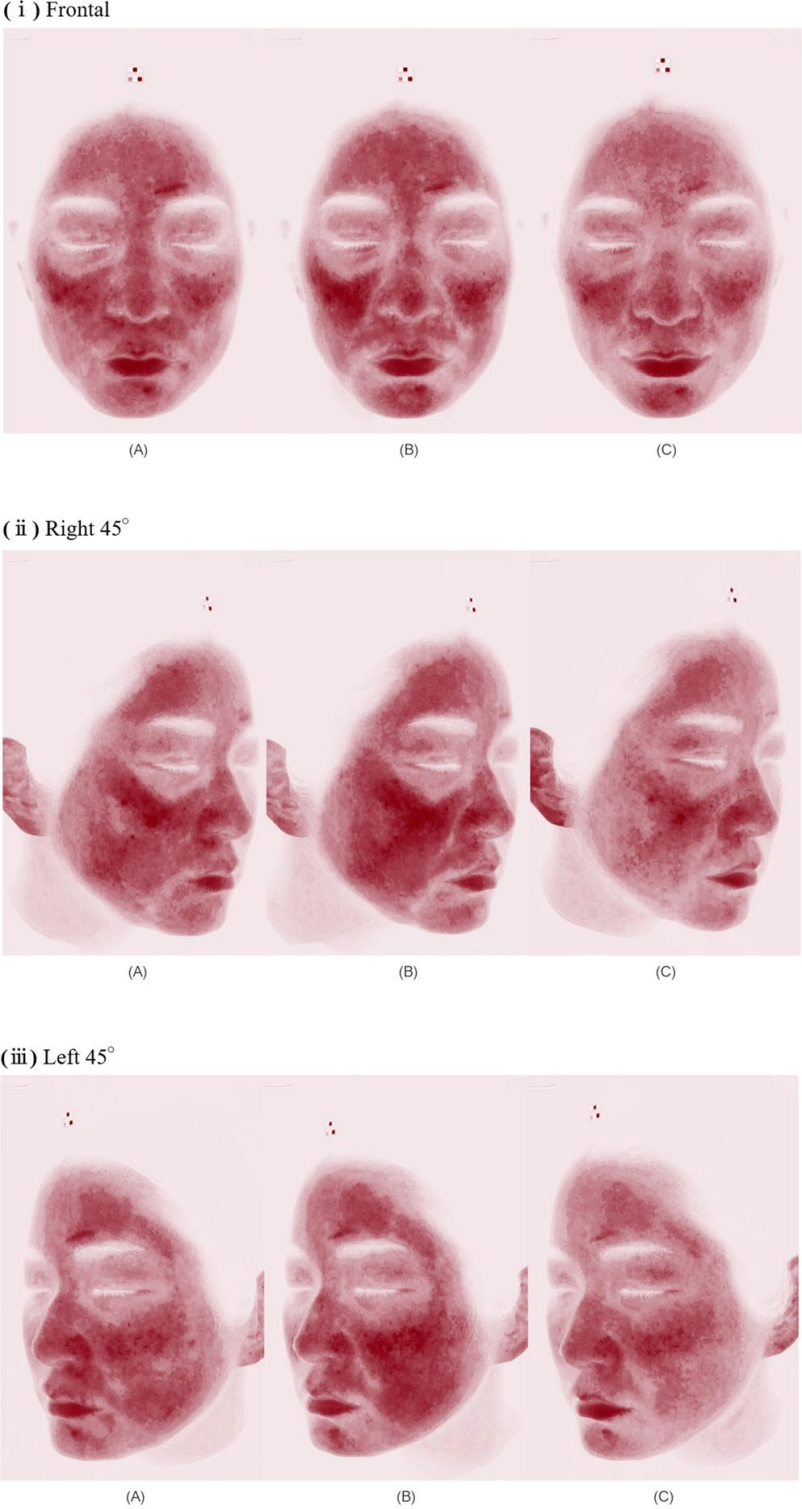
Hemoglobin-enhanced images of a 27-year-old female subject in the Pre-treatment group. **(ⅰ)** Frontal. The right side of the face was treated with nano-sized ultrafine water clusters (UFW). Erythema peaked at 1 h post-laser treatment. At 6 h, significant reduction in erythema was observed on the UFW-treated side (Rt). (**A**) Immediately after the Fr-PSAL irradiation. (**B**) 1-hour after the Fr-PSAL. (**C**) 6-hour after the Fr-PSAL. (ⅱ) Right 45°.The right side of the face was treated with nano-sized ultrafine water clusters (UFW). Erythema peaked at 1 h post-laser treatment. At 6 h, significant reduction in erythema was observed on the UFW-treated side (Rt). (**A**) Immediately after the Fr-PSAL irradiation. (**B**) 1-hour after the Fr-PSAL. (**C**) 6-hour after the Fr-PSAL. **(ⅲ)** Left 45°.The right side of the face was treated with nano-sized ultrafine water clusters (UFW). Erythema peaked at 1 h post-laser treatment. At 6 h, significant reduction in erythema was observed on the UFW-treated side (Rt). (**A**) Immediately after the Fr-PSAL irradiation. (**B**) 1-hour after the Fr-PSAL. (**C**) 6-hour after the Fr-PSAL

In the post-treatment group, each of the two blinded board-certified dermatologists independently identified the UFW-treated side in 11 of 12 subjects (91.7%; binomial test, *p* = 0.003). The two evaluators disagreed on two of the twelve cases, each evaluator misclassifying a different single case, consistent with occasional case-specific difficulty rather than systematic bias. Inter-rater observed agreement was 10/12 (83.3%), with Cohen’s kappa = 0.625 (95% CI 0.15–1.00), representing substantial agreement on the Landis-Koch benchmark. Representative imaging results are shown in Figs. 1 and 2. In the pre-treatment group, Evaluator 1 correctly identified the UFW-treated side in 11 of 13 subjects (84.6%; *p* = 0.011) and Evaluator 2 in 12 of 13 subjects (92.3%; *p* = 0.002), with a single discordant case between the two evaluators. Inter-rater observed agreement was 12/13 (92.3%), with Cohen’s kappa = 0.831 (95% CI 0.51–1.00), representing almost perfect agreement. Representative imaging results are shown in Figs. 3 and 4. No adverse reactions were observed in either group. Subjects reported reduced sensations of stinging and heat on the UFW-treated sides.

### Exploratory secondary outcome: semi-quantitative image analysis

Post-treatment group: the UFW-treated side demonstrated significantly greater reduction in erythema density compared to the control side (ΔDensity: UFW − 3.46 ± 1.55 vs. Control − 2.38 ± 1.70; mean difference − 1.07 ± 1.15; Cohen’s d = − 0.93; one-sided paired t-test, *p* = 0.004; Wilcoxon signed-rank test, *p* = 0.008). Image analysis correctly predicted the UFW-treated side in 10 of 12 subjects (83.3%).

Pre-treatment group: the UFW-treated side demonstrated significantly greater reduction in erythema area compared to the control side (ΔArea: UFW − 0.008 ± 0.028 vs. Control + 0.004 ± 0.029; mean difference − 0.012 ± 0.021; Cohen’s d = − 0.57; one-sided paired t-test, *p* = 0.031; Wilcoxon signed-rank test, *p* = 0.029). Image analysis correctly predicted the UFW-treated side in 10 of 13 subjects (76.9%).

In the pre-treatment group, the optimal comparison timepoint varied among subjects: five showed maximal differentiation at 1 h and eight at 6 h post-laser. A pre-specified sensitivity analysis using only the fixed 6 h timepoint yielded 7 of 13 correct predictions (53.8%; Cohen’s d = − 0.037; one-sided paired t-test, *p* = 0.448), indicating that the pre-treatment image-analysis result is in part dependent on the per-subject timepoint selection. This observation is interpreted in light of the two characteristic temporal patterns of post-laser erythema observed in this study (see Discussion, Sect.  4.3).

Results are summarized in Table 1.

**Table 1:** Summary of blinded clinical evaluation and exploratory image analysis results

Group	Method	Metric	*n*	Accuracy	Cohen’s d	Paired t (1-sided)	Wilcoxon (1-sided)
Post-Tx	Blinded evaluator	Visual	12	91.7% (11/12)	—	*p* = 0.003**	—
Post-Tx	Image analysis	Erythema density	12	83.3% (10/12)	−0.93	*p* = 0.004**	*p* = 0.008**
Pre-Tx	Blinded evaluator	Visual	13	84.6% (11/13)	—	*p* = 0.011*	—
Pre-Tx	Image analysis	Erythema area	13	76.9% (10/13)	−0.57	*p* = 0.031*	*p* = 0.029*

## Discussion

This pilot study demonstrates that nano-sized ultrafine water clusters significantly reduce erythema following fractionated picosecond alexandrite laser treatment, as evidenced by blinded clinical evaluation as the primary outcome. The exploratory semi-quantitative image analysis provided objective corroboration that was robust in the post-treatment arm, whereas in the pre-treatment arm it was more limited and dependent on per-subject timepoint selection (see Limitations); nonetheless, the concordance between the subjective clinical assessment and the objective image-based quantification in the primary, allocation-blinded outcome strengthens the overall validity of these findings.

### Mechanism of erythema reduction

Electroporation and facial masks are commonly employed for hydration and soothing after laser treatment. However, electroporation creates minute electrical pores in the skin and is therefore not completely non-invasive. Conversely, while facial masks are entirely non-invasive, their hydration effects are limited and short-lived. UFW offers a fully non-invasive, non-contact method with sustained moisturizing and barrier-restoring effects.

UFW particles consist of small water clusters with an aerosol-phase mobility diameter of approximately 1.4 nm, an order of magnitude below the ~ 50 nm intercellular spacing of the stratum corneum and comparable in scale to the ~ 5–7 nm sebaceous film thickness. This nanometer-scale cluster state is below the conventional radius range for which classical continuum thermodynamics predict aerosol stability, a regime in which other well-documented nanoscale water phenomena (e.g., the nanobubble paradox) have been shown to depart from continuum predictions [10, 11], supporting the experimental observation of stable ≈ 1.4 nm clusters by differential mobility analysis (Supplementary Material 1). Individual water molecules retain their intrinsic 1.85 D dipole moment, while the macroscopic polar response of the cluster can be reduced by dipole correlation through the hydrogen-bond network. As discussed in the Introduction, water confined between atomically flat walls at separations down to ~ 1 nm has been shown experimentally to exhibit a markedly low out-of-plane dielectric constant [1]; while that finding pertains to highly confined interfacial water and does not directly correspond to free aerosol clusters, it indicates that nanoscale structuring of water can substantially alter its macroscopic polar response, supporting by analogy the working hypothesis that nanometer-scale UFW clusters may exhibit reduced effective polarity relative to bulk water. Preliminary evidence from soft X-ray emission spectroscopy on PEDOT/PSS-derived UFW deposited on self-assembled monolayer model surfaces [13] (see also Supplementary Material 2) suggests the possible formation of specific ionic species, potentially including hydroxide (OH⁻) ions, via surface adsorption, although the precise formation mechanism remains under active investigation. Previous research using excised human skin and a three-dimensional cultured human skin model has shown that UFW application sustained increases in stratum corneum water content, decreased transepidermal water loss, and elevated ceramide NS levels with concomitant up-regulation of ceramide-biosynthesis genes [12], and topical UFW exposure has been reported to improve facial skin moisture and viscoelasticity in adult women [3]. A specific working hypothesis is that the small cluster state preserved during UFW transport may lower the energetic barrier for cluster-to-monomer transition at the lipid interface, thereby facilitating smoother monomer-level supply to the established transcellular and vapor-phase water transport pathways across the stratum corneum, rather than implying that intact water clusters traverse organized lipid bilayers as units. We emphasize that the hypothesized mechanistic pathway from cluster-state UFW to intracellular or intercellular hydration effects remains at the level of working hypothesis and requires direct demonstration in future mechanistic work.

### Differential metric sensitivity between protocols

An exploratory finding of this study is that the pre-specified primary metric reached statistical significance in the group for which its mechanistic basis had been hypothesized, while the sensitivity-analysis metric did not. In the post-treatment group, erythema density demonstrated a large effect size (Cohen’s d = − 0.93, *p* = 0.004), whereas the sensitivity analysis using erythema area yielded a non-significant result (d = − 0.14, *p* = 0.32). In the pre-treatment group, erythema area was the significant metric (d = − 0.57, *p* = 0.031), while erythema density was not (d = − 0.22, *p* = 0.23). This pattern is consistent with, but does not confirm, the mechanism-based hypothesis that the post-treatment protocol primarily attenuates the intensity of established erythematous foci, whereas the pre-treatment protocol primarily attenuates the formation of new erythematous foci at the time of photothermal injury. These interpretations should be considered hypothesis-generating and require confirmation in pre-registered studies.

### Two temporal patterns of post-laser erythema

In the pre-treatment group, the optimal comparison timepoint varied among subjects: five showed maximal differentiation at 1 h and eight at 6 h. Two characteristic temporal patterns of natural post-laser erythema have been reported clinically with Fr-PSAL. Pattern A (monotonic decline): erythema peaks at 0 h and decreases continuously; the protective effect of pre-applied UFW is expected to be most apparent at early timepoints (1 h) (Fig. 3). Pattern B (delayed flare-up): erythema is moderate at 0 h, then rises to a delayed peak at approximately 1 h before resolving; the protective effect of UFW is most apparent at later observation (Fig. 4). To verify the clinical rationale for the 0 h, 1 h, and 6 h observation schedule, we performed a post-hoc stratification in which each subject was classified by the timepoint at which the control-side (non-UFW) erythema area was maximal. Among 13 pre-treatment subjects, five (38.5%) were classified as Pattern A and five (38.5%) as Pattern B, confirming that both anticipated patterns were represented. A post-hoc stratified analysis of image-analysis sensitivity revealed a marked pattern-dependent difference: image analysis correctly identified the UFW-treated side in 100% (5/5) of Pattern B subjects compared with 60% (3/5) of Pattern A subjects. This observation is consistent with the interpretation that UFW’s prophylactic effect operates primarily by suppressing the delayed inflammatory flare: in Pattern B subjects, the flare on the UFW-treated side is clearly attenuated relative to the control side, producing a readily detectable bilateral asymmetry; in Pattern A subjects, the contralateral erythema already declines immediately after laser exposure, narrowing the temporal window for bilateral discrimination. Taken together, these findings support the mechanism-based framework described above and suggest that UFW’s prophylactic effect is most readily observable in patients whose natural erythema course exhibits a delayed flare. The per-subject best-timepoint analysis is nonetheless exploratory and requires prospective validation with a pre-registered primary timepoint.

#### Clinical relevance of the observed erythema reduction

The clinical relevance of the observed erythema reduction can be appraised on four complementary grounds. First, two independent board-certified dermatologists were able to identify the UFW-treated side with accuracies of 84.6%–92.3% on hemoglobin-enhanced images of post-Fr-PSAL erythema, indicating that the bilateral difference is detectable on standard clinical inspection rather than requiring instrumentation; the substantial-to-almost-perfect inter-rater agreement (Cohen’s kappa = 0.625 and 0.831) further supports reproducibility across observers. Second, the post-treatment effect on erythema density (Cohen’s d = − 0.93) is large on the Cohen (1988) benchmark and corresponds to a mean density reduction of approximately 1.07 arbitrary units beyond the contralateral (laser-only) change within 30 min of UFW application; the pre-treatment effect on erythema area (d = − 0.57) is medium, reflecting an absolute reduction in erythematous-pixel fraction visible to the dermatologist on structured image review. Third, subjects consistently reported reduced sensations of stinging and heat on the UFW-treated side—sensations that are among the principal patient-reported drivers of dissatisfaction after Fr-PSAL—so the subjective reduction is itself a clinically meaningful outcome complementing the objective erythema reduction. Fourth, no adverse events occurred across 25 subjects in a fully non-invasive, non-contact adjunctive intervention, which is a relevant consideration for clinical translation: even a modest erythema reduction has positive clinical value when obtainable without contraindication or risk.

### Limitations

Several limitations should be acknowledged. First, the sample size was small (*n* = 12 and *n* = 13), limiting statistical power; this study was designed as a pilot to establish proof of concept. Second, the primary outcome was evaluated by two independent board-certified dermatologists; while agreement was substantial (post-treatment kappa = 0.625) to almost perfect (pre-treatment kappa = 0.831), a larger panel of evaluators would further strengthen the generalizability of the inter-rater reliability estimate and should be pursued in confirmatory studies. Third, although the preliminary phantom experiment described in the Methods and in Supplementary Material 3 demonstrated approximately equivalent thermal dissipation between the UFW-treated condition and the clinical control condition, convective airflow cooling and evaporative cooling from wet gauze are distinct thermophysical processes; a dedicated sham device delivering warm airflow without UFW particles remains a worthwhile design improvement for future confirmatory trials. Fourth, Fitzpatrick skin types were predominantly II and III. Fifth, the semi-quantitative image analysis is exploratory; although the primary metric for each group and its mechanistic rationale were pre-specified, the per-subject best-timepoint selection in the pre-treatment group is post-hoc, and the fixed 6 h sensitivity analysis (7/13 correct, 53.8%; *p* = 0.448) indicates that the image-analysis finding in the pre-treatment group is partly dependent on this selection. Prospective pre-registration of the primary timepoint is warranted for confirmatory work. Sixth, a minority of subjects (3/13, 23.1%) showed continued rise of control-side erythema at 6 h, indicating that the 6 h observation window may have been insufficient to fully capture the temporal dynamics in all subjects; future studies should extend observation to 12–24 h. Seventh, UFW emission was delivered intermittently (1-minute-on / 1-minute-off cycle, approximately 15 min of cumulative active emission over the 30-minute application period); the observed clinical and image-analytic effects therefore correspond to this intermittent exposure profile, and it remains to be determined whether continuous delivery would yield a proportionally larger effect or whether the intermittent pattern itself contributes to the response through repeated exposure cycles. Eighth, although the two dermatologist evaluators and the image-analysis pipeline were blinded to allocation, the subjects themselves were necessarily unblinded, since they could perceive which side received the warm UFW airflow and which received the wet-gauze-and-wrap control; this absence of subject blinding is an inherent feature of the intervention rather than a design error, but it means that the patient-reported reductions in stinging and heat on the UFW-treated side must be interpreted with caution and that a differential subjective experience of the two sides during application cannot be entirely excluded as a contributor to the findings, whereas the allocation-blinded evaluator assessments and the objective hemoglobin-enhanced image analysis are not affected by subject unblinding. Finally, this study exclusively utilized a single laser wavelength (755 nm).

In conclusion, this pilot study provides the first clinical evidence that nano-sized ultrafine water clusters, generated by PEDOT/PSS conductive polymer technology, effectively reduce erythema following fractionated picosecond alexandrite laser treatment. Both blinded clinical evaluation and exploratory image analysis demonstrated significant erythema reduction, with the post-treatment group showing a large effect on erythema density (d = − 0.93, *p* = 0.004) and the pre-treatment group showing a moderate effect on erythema area (d = − 0.57, *p* = 0.031). UFW offers a fully non-invasive, non-contact adjunctive treatment with no observed adverse events. Larger confirmatory studies with sham-controlled designs and multiple blinded evaluators are warranted.

## Supplementary Information

Below is the link to the electronic supplementary material.

Supplementary Material 1

Supplementary Material 2

Supplementary Material 3

## Data Availability

The datasets generated during and/or analyzed during the current study are available from the corresponding author on reasonable request.
