# Supplementary material for: Efficacy of nano-sized ultrafine water clusters in reducing erythema following fractionated picosecond alexandrite laser treatment: a split-face, randomized, evaluator-blinded pilot study
Source: Lasers Med Sci. 2026 Jul 22;41(1):160. doi: 10.1007/s10103-026-04967-5 (PMC13391762; doi:10.1007/s10103-026-04967-5)
Supplement: Supplementary file 1 — Supplementary Material 1 [file 10103_2026_4967_MOESM1_ESM.pdf]

Analysis of the Characteristics of Ions Generated by Air Ionization Using a Drift Tube

Kenkichi Nagato*, Hiroyuki Mori**, Hiroto Katou**, Yuki Tabata**, Shinsuke Inoue**

* National Institute of Technology, Kochi College, Japan
** AISIN CORPORATION, Japan

Abstract
The characteristics of ions generated by air ionization and used for charging may have a significant impact on the measurement of particle size distributions of nanometer-sized particles in indoor environments. In this study, we analyzed the characteristics of positive and negative ions produced by ionizing laboratory air using a drift tube ion mobility spectrometer. A large difference was observed between positive and negative ions in the changes of their mobility spectra with air temperature. For positive ions, some ion peaks increased while others decreased with increasing air temperature. In contrast, the mobility spectrum of negative ions was almost unaffected by changes in air temperature.

Introduction
Nanometer-sized water droplets that can remain stable in air are referred to as fine water. Fine water released into the air has been reported to increase the water content of the stratum corneum, suppress transepidermal water loss, and achieve high moisturizing efficiency with a small amount of water. Accurate particle size measurement is essential to clarify these effects. When nanometer-sized particles are measured using DMA, charging ion characteristics strongly influence measurement accuracy. Therefore, this study investigated air ion characteristics using a drift tube ion mobility spectrometer.

Experimental Setup
Laboratory air was ionized using an Am-241 source, and fine water droplets were charged and analyzed using a nano-DMA with a Faraday cup electrometer. A drift tube ion mobility spectrometer with a 58 mm drift length and ±2.5 kV applied voltage was used to analyze ion mobility distributions.

Results and Discussion
Introduction of fine water shifted the positive ion distribution peak from 1.19 nm to 1.36–1.46 nm and increased ion current. Mobility spectra showed distinct behaviors for positive and negative ions, with temperature-dependent changes observed only for positive ions.

Conclusion
Ion mobility characteristics differ significantly between positive and negative air ions, particularly in temperature dependence. Further analysis using atmospheric pressure ionization mass spectrometry is planned to identify ion species.

References
Nishimura et al., Skin Research and Technology, 2019.
Ishida et al., 40th Symposium on Aerosol Science and Technology, 2023.

ドリフトチューブ法による空気電離イオンの特性解析

Analysis of the characteristics of the ions generated by air ionization using the drift tube

(高知高専) ○長門研吉*
((株)アイシン) 森隆行**，加藤寛人**，田端友紀**，井上慎介**

Abstract

The characteristics of air ionized ions used for charging may have a significant impact on the particle size distribution measurement of nanometer-sized particles in an indoor environment. We analyzed the characteristics of positive and negative ions produced by ionizing laboratory air using a drift tube ion mobility spectrometer. A large difference was observed between positive and negative ions in the change of mobility spectra with air temperature. For the positive ions, some ion peaks increased and others decreased with increasing air temperature. On the other hand, the negative ion spectrum was almost unaffected by the increase in air temperature.

1. はじめに

空気中でナノメートルサイズの大きさを保つ水滴を微細水と呼ぶ。空気中に放出された微細水は，1)肌の角質水分量を増加させる，2)肌からの水分の蒸発を抑制する，3)少ない水分量で高い保湿効果を得られる，など健康や美容に様々な効果が確認されており[1]，微細水発生装置の開発とその応用技術の研究が進められている。微細水が持つ効果のメカニズムを明らかにし，機能性の高い微細水を効率よく発生させる技術の開発を行うためには，微細水の粒径を正しく把握することが必要である。

ナノサイズの微粒子の粒径をDMAで計測する場合，微粒子の粒径と荷電に用いるイオンの大きさが近いために，どのようなメカニズムで微粒子の帯電が起こるのか明確になっていない場合が多い。特にイオンの特性によって帯電メカニズムや帯電効率が変化する可能性がある。また，微細水の粒径測定においては，微細水を実際に利用する室内環境下での測定が重要である。そのためには室内空気を電離して発生するイオンを荷電に用いることになるが，そのようなイオンの特性は室内の温度や湿度，さらに室内に存在する微量な気体成分の影響受ける可能性がある。本研究では，ドリフトチューブ型イオン移動度計を用いて，室内空気を電離して発生させたイオンの特性を調べた。

2. 実験装置

微細水の粒径を測定するために用いている計測システムをFig.1に示す[2]。 ^{241}Amを用いて実験室の空気を電離して生成した空気電離イオンが存在する領域に，微細水を通過させることで微細水を帯電させる。帯電した微細水をDMAに導入し分級してファラデーカップ型電流計で検出し，帯電した微細水の粒径分布を測定する。

Fig.2はドリフトチューブ型イオン移動度計の測定システムである。ドリフトチューブはステンレス製のガードリングをセラミックス製のスペーサリングを用いて互いに絶縁しながら重ねた構造になっている。ドリフトチューブの両端に高電圧を印加することによりドリフトチューブ内部に電場を作る。入り口には^{241}Amを配置し，エアーポンプを用いて取り込んだ空気を電離してイオンを発生させる。通常イオンはゲートによってドリフト領域には入れないが，短時間だけゲートを開けて，ドリフト領域を通過したイオンを高速電流アンプを用いて計測する。

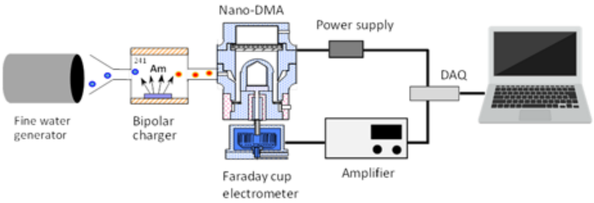

Fig.1 Measurement system of fine water using nano-DMA with Faraday cup electrometer[2].

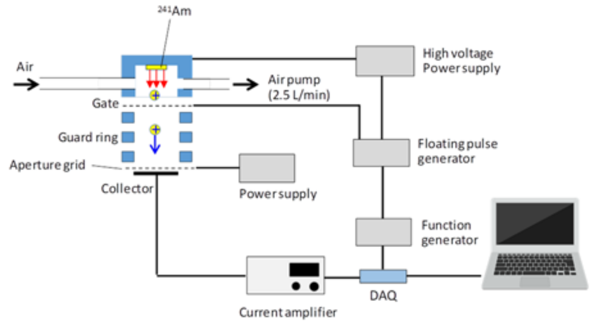

Fig.2 Schematics of the drift tube ion mobility spectrometer.

3. 実験結果

3-1. 微細水の粒径分布測定

DMAを用いて微細水を計測した結果をFig.3に示す。微細水を導入せずに空気電離正イオンを測定した結果に比べて，微細水を導入した場合は粒径分布が大きく変化した。空気電離正イオンの粒

*Kenkichi Nagato, National Institute of Technology, Kochi College, Nankoku, Kochi 783-8508, Japan
**Hiroyuki Mori, Hiroto Katou, Yuki Tabata, Shinsuke Inoue, AISIN CORPORATION, Kariya, Aichi 448-8650, Japan

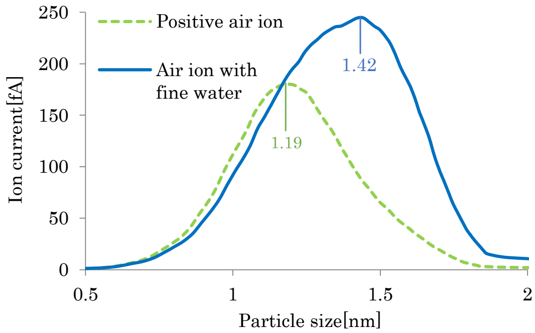

Fig.3 Positive ion size distributions of laboratory air and of laboratory air with fine water.

径分布は1.19 nmを中心とした分布となったが，微細水を導入すると分布のピークが1.36-1.46 nmの範囲へと増大し，イオン電流のピークも180 fAから244 fAへ増加した。

3-2. 空気電離イオンの移動度スペクトル

空気電離によって生成した正および負イオンの移動度スペクトルをドリフトチューブを用いて測定した結果をFig.4に示す。ドリフト領域の長さは58 mm，印加電圧は±2.5 kVで，イオンのドリフト時間は約7-16 msである。

負イオンは1.94 $cm^2V^{-1}s^{-1}$に大きなイオンピークが観測される。単一のピークのように見えるが，近接した2つのイオンピークが重なり合っている可能性がある。一方，正イオンは1.8-0.8 $cm^2V^{-1}s^{-1}$の範囲に幅広く分布している。その範囲に1.71 $cm^2V^{-1}s^{-1}$，1.44 $cm^2V^{-1}s^{-1}$，1.34 $cm^2V^{-1}s^{-1}$の3つのイオンピークが存在していることがわかる。イオン電流は負イオンの方が大きいが，正イオンに比べてドリフト時間が短いために，ドリフト領域を移動する間の拡散による消失が正イオンよりも少ないためであると考えられる。

3-3. 空気電離イオン移動度スペクトルの温度変化

ドリフトチューブの空気導入部のステンレス配管にリボンヒーターを巻いて加熱し，導入する空気温度を上昇させた場合の正イオンおよび負イオンの移動度スペクトルの変化をそれぞれFig.5とFig.6に示す。正イオンスペクトルでは空気温度が18.9℃から39.6℃まで上昇する間に，3つのイオンピークの強度に変化が観測された。移動度の大きな2つのピークは空気温度の上昇とともにイオン強度が増加したが，一番移動度が小さなピークは逆に減少した。一方，負イオンのスペクトルには，空気温度による変化が全く観測されなかった。

4. おわりに

空気電離イオンの移動度スペクトルをドリフトチューブを用いて計測し，その特性の分析を行っている。空気の温度による影響は正イオンと負イオンでは大きく異なった。空気の湿度や室内の様々な発生源によるVOCの影響を受けることも明らかになりつつある。このようなイオン特性の原

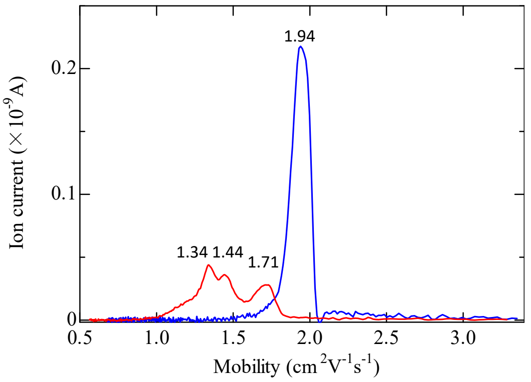

Fig.4 Mobility spectra of positive and negative ions generated by ionization of laboratory air.

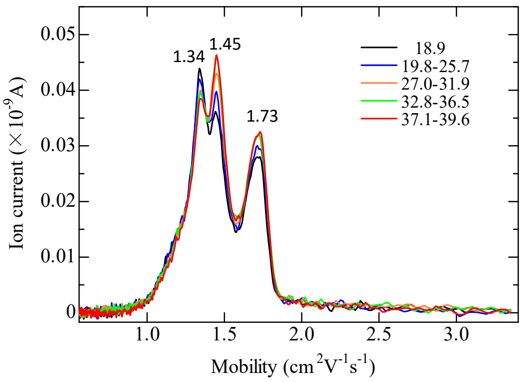

Fig.5 Mobility spectral change of positive ions with increasing air temperature.

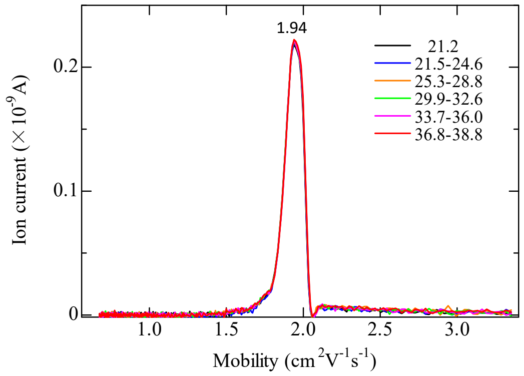

Fig.6 Mobility spectral change of positive ions with increasing air temperature.

因を解明するために，大気圧イオン化質量分析計によるイオン質量スペクトルの測定も同時に進めていく予定である。

References

1) Nishimura *et al.*, (2019), Effect of spraying of fine water particles on facial skin moisture and viscoelasticity in adult woman, Skin Research and Technology, 25, 294-298
2) 石田他，(2023)，DMAによるナノサイズ微細水の粒径計測，第40回エアロゾル科学・技術討論会
