# Supplementary material for: Efficacy of nano-sized ultrafine water clusters in reducing erythema following fractionated picosecond alexandrite laser treatment: a split-face, randomized, evaluator-blinded pilot study
Source: Lasers Med Sci. 2026 Jul 22;41(1):160. doi: 10.1007/s10103-026-04967-5 (PMC13391762; doi:10.1007/s10103-026-04967-5)
Supplement: Supplementary file 2 — Supplementary Material 2 [file 10103_2026_4967_MOESM2_ESM.pdf]

NANOBUBBLE 2022, Conference on Nanobubbles, Nanodroplets and their Applications,

September 18-21, 2022, Festung Mark, Magdeburg, Germany

Electronic structure of ultrafine water cluster deposited on hydrophobic and hydrophilic surfaces explored by soft X-ray emission spectroscopy

Yoshihisa Harada[1,2,*], Ayako Kameda[2], Ralph Ugalino[2], Naoya Kurahashi[1], Hisao Kiuchi[1,2], Tomohiro Hayashi[3], Yuki Tabata[4], Akiyoshi Hirano[4], Shinsuke Inoue[4]

1 *Institute for Solid State Physics, The University of Tokyo, 5-1-5, Kashiwanoha, Kashiwa, Chiba 277-8581, Japan*
2 *Department of Advanced Materials Science, Graduate School of Frontier Sciences, The University of Tokyo, 5-1-5, Kashiwanoha, Kashiwa, Chiba 277-8561, Japan*
3 *Department of Materials Science and Engineering, School of Materials Science and Chemical Technology, Tokyo Institute of Technology, 4259, Nagatsuta-cho, Midori-ku, Yokohama, Kanagawa 226-8503, Japan.*
4 *AISIN CORPORATION, 2-1, Asahi-cho, Kariya, Aichi 448-8650, Japan*

E-Mail corresponding author: harada@issp.u-tokyo.ac.jp

Abstract

In recent years, water clusters called ultrafine water which has several nanometers or less have attracted attention as having a wide range of functions such as humidification, moisture retention, deodorization, air cleaning and sterilization, and static electricity removal. However, little is known about the mechanism of their functions. For example, in moisturizing, penetration into the subcutaneous tissue is considered because the ultrafine water is smaller than the intercellular space on the skin surface. However, this alone cannot account for the long-lasting moisturizing effect [1]. Therefore, it is necessary to clarify the physicochemical properties of ultrafine water to understand those macroscopic functions. In this study, we investigated the electronic structure of ultrafine water deposited on hydrophobic and hydrophilic surfaces using O 1s X-ray emission spectroscopy (XES) to obtain detailed information on the hydrogen-bonded configuration of ultrafine water on those surfaces, and to discuss the relationship between the chemical state and the function of ultrafine water. Figure 1 shows a schematic of the XES process for water. The incident soft X-ray excites a core electron to an unoccupied state (XAS: Fig. 1a), and another soft X-ray emits in the subsequent decay of the core hole (XES: Fig. 1b). When the valence electron decays back to the core hole, XES will represent occupied valence electronic structures of water, which is quite sensitive to various hydrogen-bonded configurations in water [2]. Figure 2 illustrates the experimental setup for

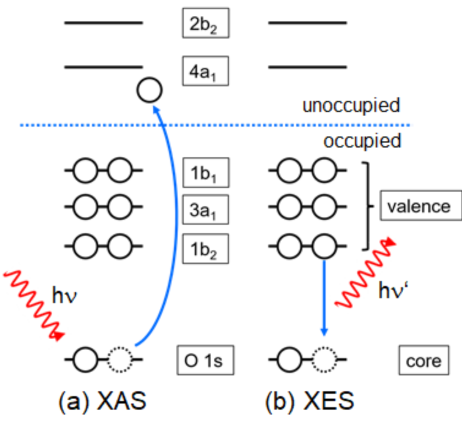

Figure 1 Schematic of the (a) XAS and (b) XES processes for water

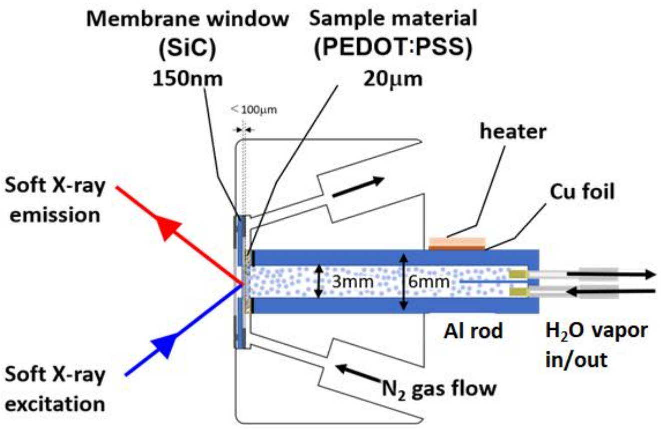

Figure 2 Setup for XES measurement of ultrafine water

the XES measurement. By repeating a heating (by a ceramic heater up to 60 °C) and fully humidifying cycle on a poly(3,4-ethylenedioxythiophene) polystyrene sulfonate (PEDOT:PSS) membrane, ultrafine water of the size of c.a. 1.4 nm is generated. The emitted ultrafine water are deposited on hydrophobic (CF_3: HS-$(CH_2)_{11}$-(O-CH_2-CH_2)-$(CF_2)_5$-CF_3) or hydrophilic (EG_3OH: HS−$(CH_2)_{11}$−(O−CH_2−$CH_2)_3$−OH) self-assembled monolayer (SAM) coated silicon carbide thin membrane which separates vacuum from the atmosphere and effectively transmits incoming and outgoing soft X-rays. The energy dispersion of the emitted soft X-rays is analyzed by the soft X-ray emission spectrometer HORNET at BL07LSU [3] in SPring-8.

The O 1s XES spectrum of ultrafine water on a hydrophobic (CF_3-SAM) surface is shown in Figure 3a. Prior to be installed in a measurement cell, PEDOT:PSS was heat-treated for 15 min. at about 330K for degassing. The CF_3-SAM surface was humidified for approximately 9.5 h with ultrafine water, and the integrated XES spectrum is depicted in blue. In red is a dry spectrum acquired by passing N_2 gas through the measurement cell. The difference between the blue and red spectra generated the black spectrum, which is very similar to that of H_2O gas, apart from a tiny change in the $3a_1$ region.

Figure 3b illustrates the O 1s XES spectra of ultrafine water on the CF_3-SAM exposed to air, where the PEDOT:PSS was not heat-treated prior to the measurement. By exposing the CF_3-SAM to oxygen moieties released from the PEDOT:PSS, the CF_3-SAM should become partly hydrophilic. During the initial 216 min., the XES profile remained unchanged, but subsequently began to grow the $1b_1$' peak around 526eV. The black XES spectrum was derived by subtracting the integrated spectra before 216 min. from those after 216 min. Only the two small peaks indicated by the arrows deviate from the spectrum obtained by using a conventional humidifier which deliver 10μm-order water droplets (not shown).

We speculate that these two peaks come from specific ionic species, such as OH^- ions [4]; their sharpness indicates that OH^- adsorption occurs at a specific surface site. The fact that ultrafine water forms ions via surface adsorption may explain why it is good for skin moisturization, albeit the method by which OH^- ions are formed is still being debated.

In the presentation we will also present results for the hydrophilic EG_3OH-SAM surface and discuss possible difference in interaction of ultrafine water with the hydrophobic and hydrophilic surfaces.

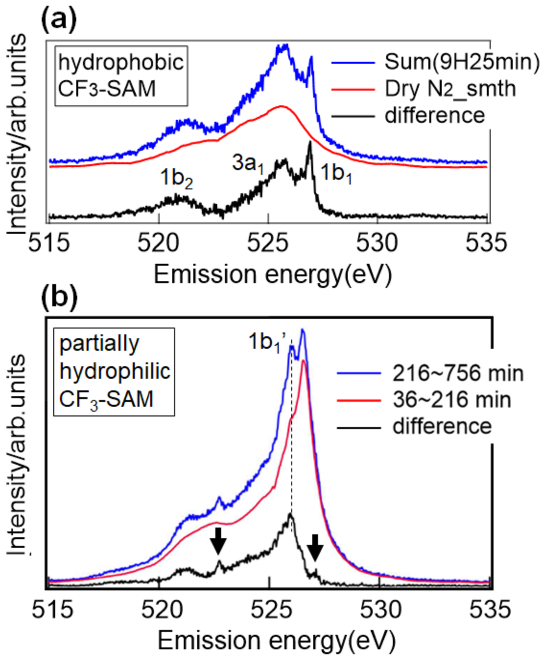

Figure 3 O 1s XES of water on (a) hydrophobic and (b) partially hydrophilic CF_3-SAM. Blue: raw data, Red: background, Black: background subtracted.

Acknowledgements

This study was supported by JSPS KAKENHI grant number JP19H05717 (Grant-in-Aid for Scientific Research on Innovative Area: Aquatic Functional Materials).

References

[1] N. Nishimura *et al.*, Effect of spraying of fine water particles on facial skin moisture and viscoelasticity in adult women, *Skin Res. Technol.* **2019**, *25*, 294.

[2] T. Tokushima *et al.*, High resolution X-ray emission spectroscopy of liquid water: The observation of two structural motifs, *Chem. Phys. Lett.* **2008**, *460*, 387.

[3] Y. Harada *et al.*, Ultrahigh resolution soft x-ray emission spectrometer at BL07LSU in SPring-8, *Rev. Sci. Instrum.* **2012**, *83*, 013116.

[4] O. Fuchs *et al.*, Isotope and Temperature Effects in Liquid Water Probed by X-Ray Absorption and Resonant X-Ray Emission Spectroscopy, *Phys. Rev. Lett.* **2008**, *100*, 027801.
