# Supplementary material for: Efficacy of nano-sized ultrafine water clusters in reducing erythema following fractionated picosecond alexandrite laser treatment: a split-face, randomized, evaluator-blinded pilot study
Source: Lasers Med Sci. 2026 Jul 22;41(1):160. doi: 10.1007/s10103-026-04967-5 (PMC13391762; doi:10.1007/s10103-026-04967-5)

Selection of Control Conditions

Configuration Overview
and Experimental Photographs

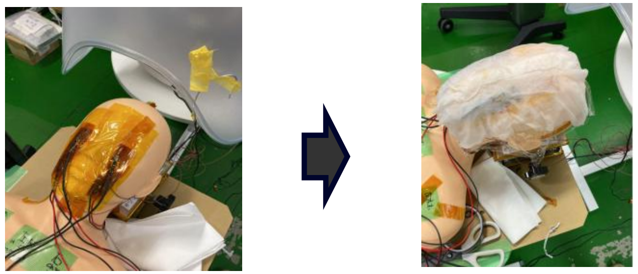

Pre-treatment Group

Contralateral Side

Reference: Wrap Only

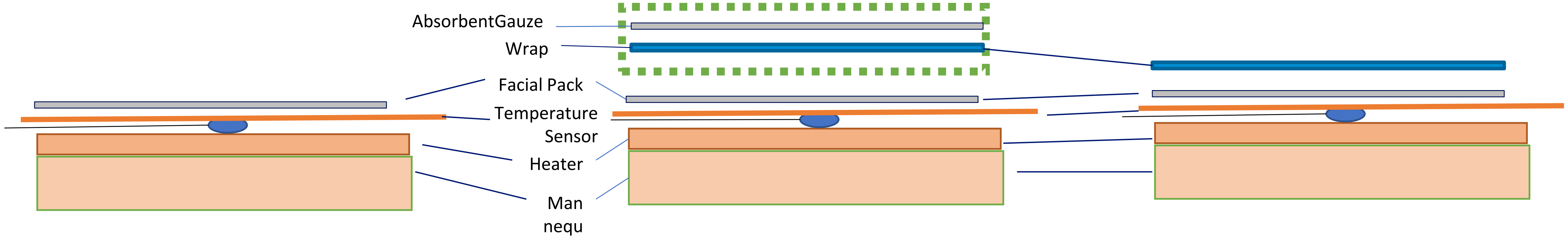

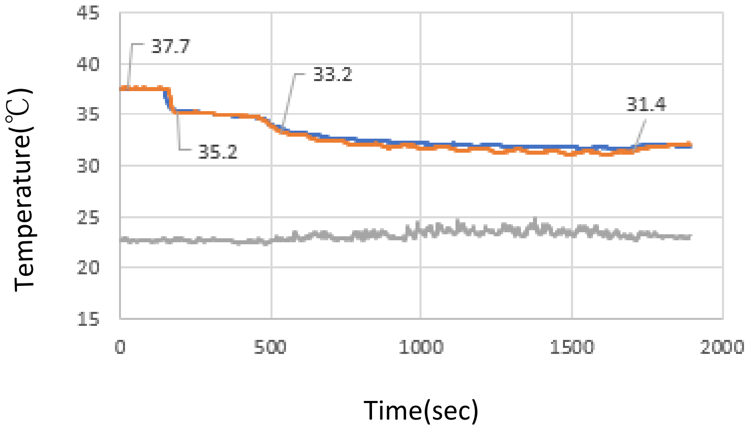

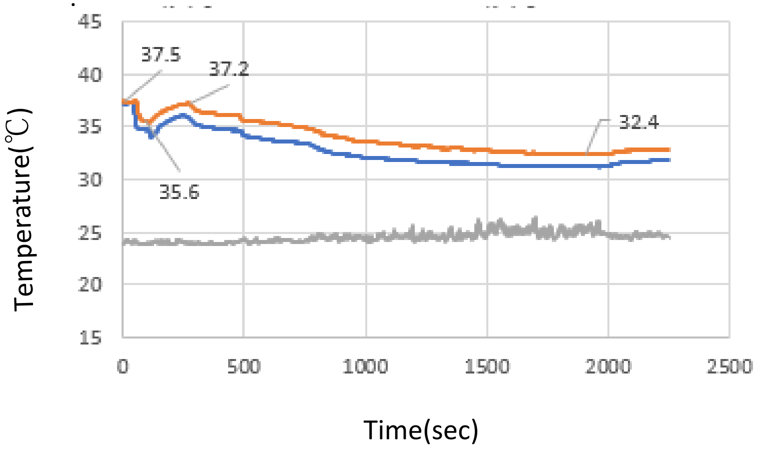

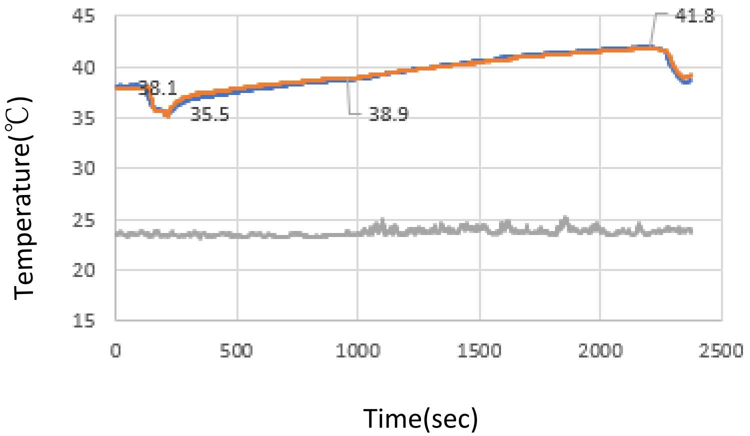

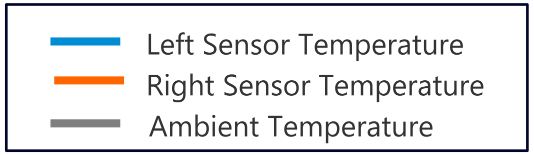

Supplement: Supplementary file 3 — Supplementary Material 3 [file 10103_2026_4967_MOESM3_ESM.pdf]
